# Development of transgenic wheat (*Triticum aestivum* L.) expressing avidin gene conferring resistance to stored product insects

**DOI:** 10.1186/s12870-015-0570-x

**Published:** 2015-07-22

**Authors:** Heba H Abouseadaa, Gamal H Osman, Ahmed M Ramadan, Sameh E Hassanein, Mohamed T Abdelsattar, Yasser B Morsy, Hussien F Alameldin, Doaa K El-Ghareeb, Hanan A Nour-Eldin, Reda Salem, Adel A Gad, Soheir E Elkhodary, Maher M Shehata, Hala M Mahfouz, Hala F Eissa, Ahmed Bahieldin

**Affiliations:** Department of Botany, Faculty of Science, Ain Shams University, Cairo, Egypt; Agricultural Genetic Engineering Research Institute (AGERI), Agriculture Research Center (ARC), Giza, 12619 Egypt; Department of Biology, Faculty of Applied Sciences, Umm Al Qura University, Makkah, 21955 Saudi Arabia; Department of Biological Sciences, Faculty of Science, King Abdulaziz University (KAU), Jeddah, 80203 Saudi Arabia; Plant Soil and Microbial Sciences Department, Michigan state University, East Lansing, M9, 48824 USA; College of Biotechnology, Misr University of Science and technology, Giza, Egypt; Department of Genetics, Faculty of Agriculture, Ain Shams University, Cairo, Egypt

**Keywords:** Stored cereals insects, post harvest loss, avidin, transgenic wheat

## Abstract

**Background:**

Wheat is considered the most important cereal crop all over the world. The wheat weevil *Sitophilus granarius* is a serious insect pests in much of the wheat growing area worldwide and is responsible for significant loss of yield. Avidin proteins has been proposed to function as plant defense agents against insect pests.

**Results:**

A synthetic *avidin* gene was introduced into spring wheat (*Triticum aestivum* L.) cv. Giza 168 using a biolistic bombardment protocol. The presence and expression of the transgene in six selected T_0_ transgenic wheat lines were confirmed at the molecular level. Accumulation of avidin protein was detected in transgenic plants compared to non-transgenic plants. Avidin transgene was stably integrated, transcribed and translated as indicated by Southern blot, ELISA, and dot blot analyses, with a high level of expression in transgenic wheat seeds. However, no expression was detected in untransformed wheat seeds. Functional integrity of avidin was confirmed by insect bioassay. The results of bioassay using transgenic wheat plants challenged with wheat weevil revealed 100 % mortality of the insects reared on transgenic plants after 21 days.

**Conclusion:**

Transgenic wheat plants had improved resistance to *Sitophilus granarius*.

## Background

Wheat is the first most important field crop worldwide in terms of crop value and total production. Wheat production is limited by both biotic and abiotic factors [1]. Insect infestations are major factors for post harvest loss of grain quantity and quality. Preventing or at least slowing the stored product infestation is important in maintaining wheat’s quality and marketable volume [2]. Insect pests contaminate stored cereals and cause damage by their feeding, producing highly toxic and carcinogenic compounds, and creation of allergens. Additionally, their metabolic products change the smell and taste of the contaminated baking products [3]. There is an urgent need to eliminate wheat insect infestation in stores, during transportation and processing, which ensures a supply of wholesome food to the consumers [4]. Chemical insecticides are being used to reduce the negative impact of the transmission of the insect infestations [5]. Complete dependency on the heavy use of chemicals has created numerous unacceptable agricultural, environmental and human health problems. Another concern is the development of resistance in target organisms [6,7]. These factors have encouraged the scientific community to discover alternative, bio-friendly, economically acceptable strategies for insect control [8–10]. Considerable progress has been made over the past two decades in manipulating genes from diverse sources and inserting them into crop plants to confer resistance to insect pests, diseases, tolerance to herbicides, drought, improved nutritional quality, increased effectiveness of bio-control agents, and a better understanding of the nature of gene action and metabolic pathways [11–14]. Transgenic biotechnology can be utilized as an alternative choice for protection of crops from attack by insect pests using insect growth-inhibiting proteins e.g., insect chitinase [15], cry protein [16], vip3A protein [17] and avidin protein [18]. If genes coding for these proteins are introduced into wheat with adequate and stable expression, they can provide resistance against stored product pests over several generations. Avidin is a glycoprotein that binds biotin strongly and prevents acquisition of biotin by many organisms [19]. Biotin is a cofactor needed during important carboxylation reactions. Insects have no biosynthetic pathway for biotin and thus, must obtain it from other sources. Therefore, diets containing avidin are toxic to a wide range of insects [20]. The molecular weight of avidin is about 67 kDa. The insecticidal effect of chicken avidin has been known since 1959 when it was first reported that the protein is toxic to the housefly, *Musca domestica* [21], this effect is due to the strong binding of avidin to biotin [22]. Due to its insecticidal properties, avidin has been expressed in a variety of agriculturally important plant species, for example, tobacco, maize and rice [18,19]. Avidin differs from other transgenic insecticidal toxins because it is not directly damaging to tissues, rather it merely withholds an essential nutrient from the insects [23]. The biotin binding activity of avidin is greatly destroyed by cooking, rendering the avidin harmless to humans following cooking. In the same way that cooked eggs (or precisely egg white) are not harmful to humans and are considered as a normal component of many people's diet [24]. Ninety seven percent of avidin's functional activity is lost by heat denaturation (i.e. cooking) at 95 °C for 5 min. In addition, avidin has the considerable advantage over conventional insecticides that it is not washed away during processing and continues to act as an insecticide during storage [20]. In this study, we introduced a modified *avidin* gene into wheat in order to protect it from stored product insects, and we investigated the insecticidal activity of transgenic wheat against wheat weevil.

## Methods

### Insect

Wheat weevil *Sitophilus granarius* were obtained from the insectary at the Agricultural Genetic Engineering Research Institute (AGERI) - ARC- Egypt.

### Construction of recombinant genes

**Fig. 1:**
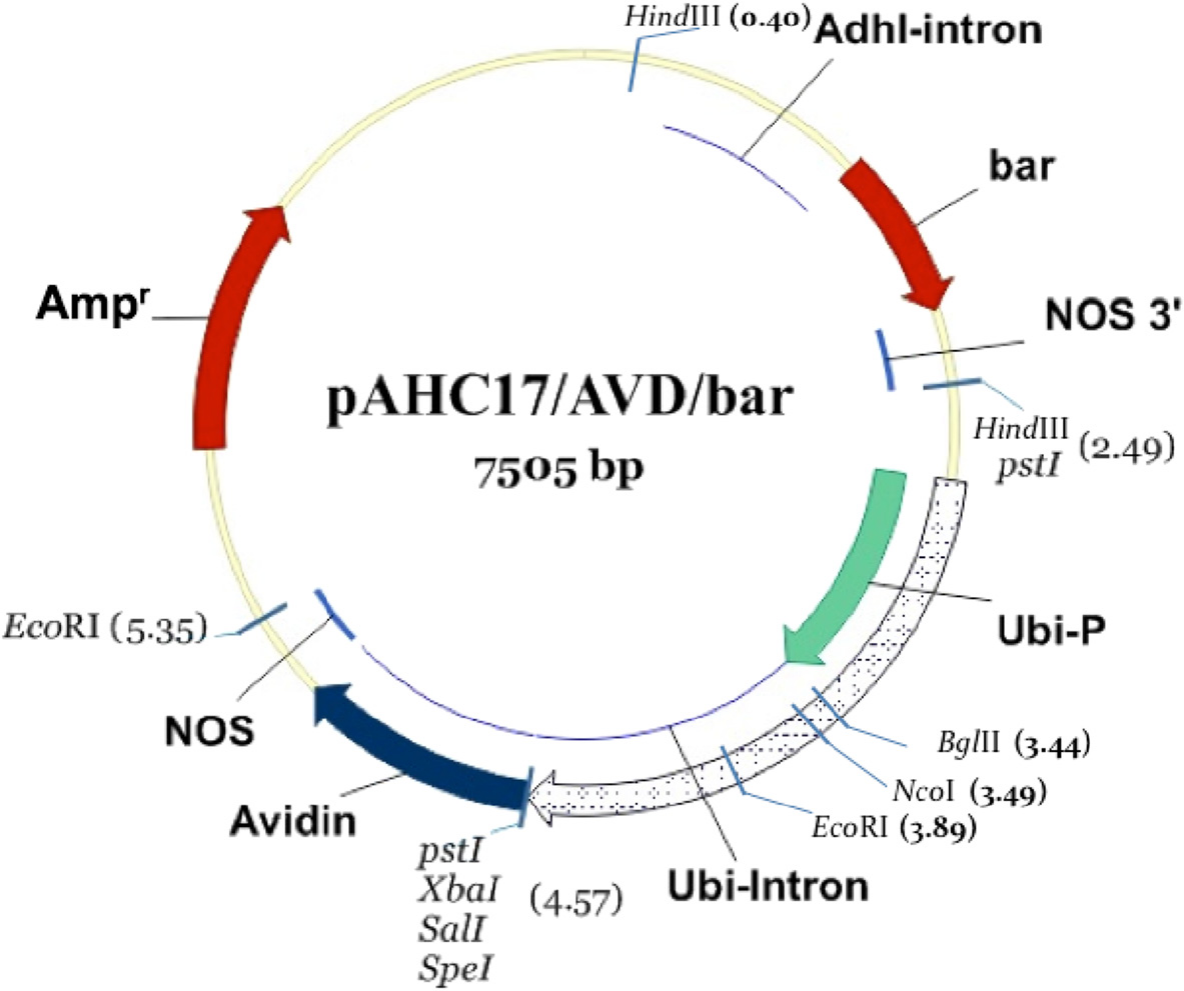
The plant expression vector pAHC17/avidin/bar

The synthetic avidin gene including Barley alpha amylase signal peptides was designed as outlined by [20]. Codon bias was checked through the codon usage table of wheat (NCBI-GenBank, http://www.kazusa.or.jp/codon). An 496 bp *EcoRI* fragment containing the full-length synthetic avidin (synthesized by Bioneer co., http://eng.bioneer.com) was filled using Klenow fragment and blunt-end ligated to the previously digested and filled *BamHI* site of pAHC17 [25].The 2100 bp *HindIII* fragment containing *bar* gene cassette with the ubiquitin promoter was at the *HindIII* site of pAHC17 (Fig. 1).

### Wheat transformation

Immature embryos were isolated from field grown bread wheat (*Triticum aestivum* L.) cv. Giza 168(G168).Tissue culture and transformation systems were performed [26]. Primary transformants were transferred to the biocontainment facility and tested using leaf painting assay with a 0.1 % aqueous solution of Basta™ (Bayer Crop Science PVT Ltd) containing 20 % glufosinate ammonium.

### Polymerase chain reaction (PCR)

Genomic DNAs were extracted from putative transgenics plants, resistant to the herbicide Basta, as well as the wild type control, using the CTAB method [27]. PCR was performed by the amplification of *avidin* gene (496 bp) as well as for the *bar* gene (400 bp) using specific primers (Table 1).

**Table 1:** Avidin and bar gene amplification primers

No	Primer Name	Primer Sequence	Amplification product Size (bp)
1	*bar* (forward)	5^׳^ TAC ATC GAG ACA AGC ACG GTC AAC T 3^׳^	400
	*bar* (reverse)	5^׳^ ACG TCA TGC CAG TTC CCG TG 3^׳^	
2	*avidin* (forward)	5^׳^GAA TCC ATG GCT AAC AAG CAC CTC AGC CTG 3^׳^	496
	*avidin* (reverse)	5^׳^CCT AGG TCA CTC CTT CTG GGT CCT CAG TCT T 3^׳^	

The reaction conditions were optimized and mixtures (50-μl total volume) composed of dNTPs (0.2 mM), MgCl_2_ (1.5 mM), 1x buffer, primer (0.2 μM), DNA (100 ng), *Taq* DNA polymerase (2 units). Amplification was carried out in a Biometra^TM^ PCR programmed for 40 cycles as follows: 94 °C/4 min for primary denaturation (1 cycle); 94 °C/1 min for denaturation, 55 °C/1 min for annealing with *bar* gene primers and 58 °C/1.2 min for annealing with *Avidin* gene primers, 72 °C/2 min for extension (38 cycles); 72 °C/8 min (1 cycle); 4 °C (overnight storage). Agarose (1.2 %) was used for resolving PCR products. Gene Ruler 100 bp DNA ladder (Fermentas, USA.) was also run in the same gel. The run was performed at 80 V in Bio- Rad submarine (8 cm X 12 cm) electrophoresis unit. Bands were detected on a UV-transilluminator and photographed.

### Southern blot analysis

Genomic Southern blot analysis [28] was carried out using DIG High Prime DNA Labeling and Detection Starter Kit II (Roche cat. No. 11 585 614 910) for the six selected T_0_ transgenics. Genomic Southern analysis of the six avidin transgenic plants AVD1, AVD2, AVD3, AVD4, AVD5 and AVD6 . Genomic DNA of each line was digested with *NcoI* and *SpeI* and fragmented by 0.8 % agarose gel electrophoresis. The blot was probed with a *SpeI* and *HindIII* fragment involving a maize ubi intron1, maize ubi promoter. Ubi is 2.08 kb.

### RT-PCR

Total RNA was extracted from transgenic as well as control non-transgenic plants using total RNA isolation system (Trizol reagent - Sigma-Aldrich, USA). Expression of the integrated transgene was tested on the RNA extracted from T_1_ plants that showed positive results after being sprayed with the Basta herbicide using a semi-quantitative RT-PCR according to the protocol described by Eltayeb et al., [29]. RT-PCR was performed with RevertAid H Minus First Strand cDNA Synthesis Kit (Thermo Scientific ^TM^, cat. No. K1631) using *avidin* and actin reverse primers, separately. The reactions were followed with PCR reaction using GoTaq Flexi DNA polymerase (Promega, USA) identical to those used for the PCR analysis to generate an expected product size of 496 bp. was used as a house keeping gene to normalize the initial variations in sample concentration. *Actin* was amplified using forward primer: 5' TGA CGT GGA TAT CAG GAA GG 3' and reverse primer 5' GCT GAG TGA GGC TAG GAT GG 3' to generate expected product at196 bp, and the *avidin* primer was used as mentioned above. PCR conditions were: initial denaturation at 94 °C for 3 min; 40 cycles of 94 °C for 10 s, 58 °C for 30 s and 72 °C for 15 s; elongating at 72 °C for 10 min.

### ELISA and dot blot analysis

ELISA performed with average of four replicates per samples according to the procedure of Clark *et al.,* [30] and Dot blot analysis was performed as outlined in Gil et al., [31] to confirm the presence of the avidin protein in the grains of the T_2_ transgenic plants using anti-avidin rabbit whole serum (Sigma, USA, Cat NO. A5170). Two μg of avidin protein (Sigma-Aldrich cat. A9275-1MG) was used as a positive control.

### Insects Bioassay

An insect feeding bioassay experiment was conducted according to the method described earlier [19,20] using three replicates of crushed seeds of transgenics and non- transgenic plants. Every replicate contained 100 mature insects of *Sitophilus granarius* for 21 days at 25–30 °C. Bioassay was repeated 3 times and mortality was scored daily until death or pupation.

## Results

This study aimed to introduce the synthetic *avidin* gene into immature embryo-derived calli of an Egyptian hexaploid bread wheat (spring wheat cv. Giza 168), and to investigate its ability to confer insecticidal effect on wheat plants *via* microprojectile bombardment using a eukaryotic expression vector containing the *bar* gene as the selectable marker. The modified *avidin* gene sequence that was used in our transformation experiments was synthesized according to a report [20] where the same sequence in the transformation of rice (*Oryza sativa*) plants was used.

### Molecular Analysis

#### Construction of the plant expression vector pAHC17/*avidin*/*bar* harboring the *avidin* gene

The plant expression vector used in this study was pAHC17/*avidin*/*bar* harboring the modified a*vidin* gene [20] under the control of *Zea mays ubiquitin* promoter and *bar* gene under the control of CaMV 35S promoter.

**Fig. 2:**
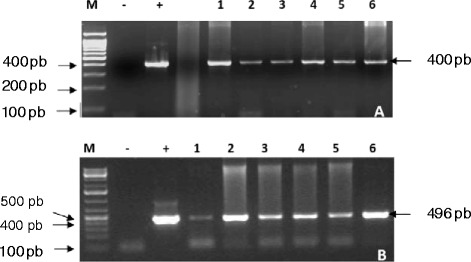
PCR product of (**a**) partial-length *bar* gene and (**b**) full-length *avidin* gene of the six independent transgenic lines. (1–6; transgenic events AVD 1, AVD 2, AVD 3, AVD 4, AVD 5 and AVD 6). M; GeneRuler DNA ladder (Fermentas, USA.), +; positive control pAHC17/*avidin*/*bar*, −; negative control (non-transgenic) cv., Giza 168

PCR was performed using specific primers of the two transgenes (*bar* and *avidin*) using DNA extracted from the T_0_ transgenic plants that were scored for resistance to the Basta herbicide, in order to confirm the presence of both genes in their genomic DNA. From the results reported in Fig. 2a and b, both transgenes are present in the genomic DNA of the six putative transgenic plants. Figure 2a shows the PCR product corresponding to the expected size of the partial-length *bar* gene amplification product (400 bp) and Fig. 1b shows the PCR product corresponding to the expected size of the full-length *avidin* gene (496 bp).

### Southern blot analysis

**Fig. 3:**
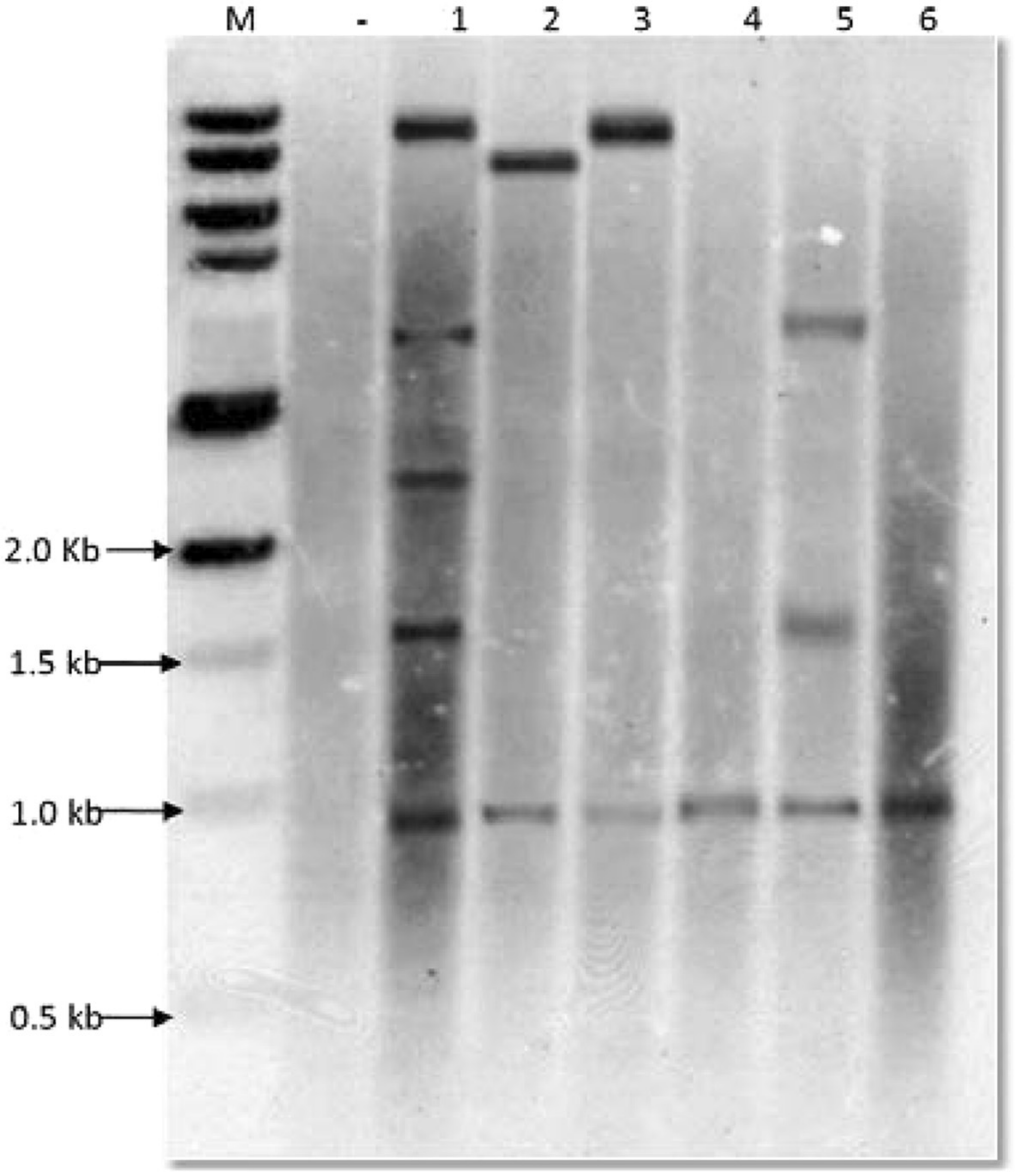
Genomic Southern blot analysis of the six avidin transgenic plants AVD1, AVD2, AVD3, AVD4, AVD5 and AVD6 (lane 1–6, respectively). Genomic DNA of each line was digested with *NcoI* and *SpeI* and fragmented by 0.8 % agarose gel electrophoresis. The blot was probed with a *SpeI* and *HindIII* fragment involving a maize Ubi intron 1and maize ubi promoter, ubi (2.08 kb). M, 1 Kb NEB DNA ladder (0.5, 1, 1.5, 2, 3, 4, 5, 6, 8, 10 kb), *HindIII*-digested DNA of non-transgenic Giza 168 cultivar

**Fig. 4:**
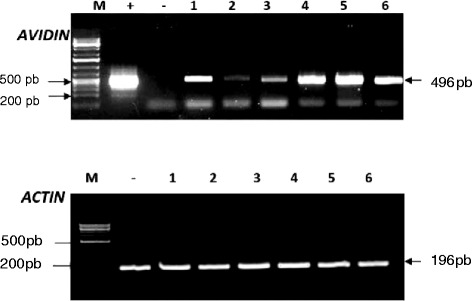
SqRT-PCR involving *avidin* gene for the six transgenic plants , (+) avidin plasmid and (−) negative control plant. (1–6; transgenic events AVD1, AVD2, AVD3, AVD4, AVD5 and AVD6). (−)negative control Giza 168, and *actin* gene was used as the reference gene for relative amount of RNA. M, GeneRuler 1 Kb DNA Ladder (Fermentas,USA)

Total genomic DNA isolated from leaf tissue of plants that tested positive in the leaf painting assay with the herbicide and these that tested positive in PCR analyses of putative transgenics as well as the non-transgenic wheat cultivar Giza 168 as negative control were digested with the restriction enzyme mixture of *SpeI* and *NcoI*. The digestion products were subjected to electrophoresis followed by southern blot analyses and hybridized with the *HindIII*/*SpeI* restriction fragment (2.08 kb) of the pAHC17/avidin/bar plasmid, which was used as a probe to confirm the integration of pAHC17/avidin/bar construct in the wheat genome. Figure 3 shows the hybridization patterns of the probe with genomic DNA of the six putative transgenics (AVD1, AVD2, AVD3, AVD4, AVD5 and AVD6) and the negative control (G 168). As expected, a fragment with expected size (~1 kb) was detected from genomic DNA of the six transgenics and was completely absent from the non-transgenic wheat cultivar (G 168). Different insertion sites patterns (variable bands sizes per well) confirms that the six transgenic events were independently originated from multiple independent embryogenic calli representing different integration events. In addition, the numbers and sizes of bands indicated different copy numbers of the transgene avidin. For example AVD1 has four copies and AVD5 has two copies and AVD2 has one copy. Semi-quantitative Reverse transcriptase-polymerase chain reaction (SqRT-PCR) analysis: Expression of the integrated gene was tested on the RNA extracts of the T1 plants that showed positive results after being sprayed with the Basta herbicide. The results of avidin gene expression indicated the presence of the expected cDNA band size 496 bp (Fig. 4). SqRT-PCR detected differential expression levels between the six transgenic lines. The transcription level of avidin in the AVD1, AVD4, AVD5 and AVD6 is shown to be relatively higher than those of AVD 2 and AVD3 lines.

### Protein Analysis

#### Protein dot blot assay

**Fig. 5:**
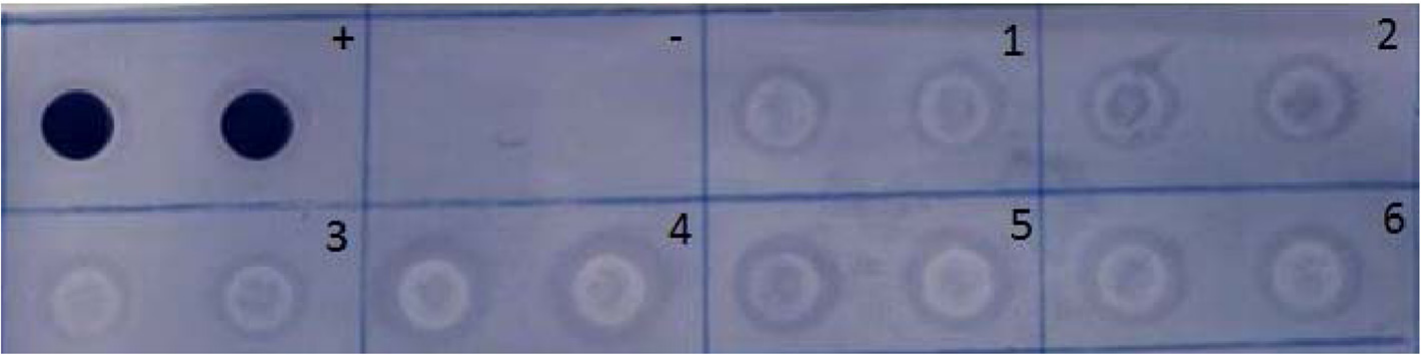
Dot blot analysis of the avidin protein in the grains of the six transgenic plants and (+) positive control avidin crude protein from egg white (Sigma, A9275), (−) negative control (non-transgenic) cv., Giza 168. (1–6) AVD1, AVD2, AVD3, AVD4, AVD5 and AVD6

Avidin protein in the grains of transgenic plants, which were resistant to the Basta herbicide and Giza 168 non-transgenic cultivar was used as negative control. The six transgenic lines AVD1, AVD2, AVD3, AVD4, AVD5 and AVD6 (Fig. 5) expressed the avidin protein as well as the positive signal of the authentic *avidin*, while the non-transgenic cultivar, Giza 168, showed negative signal.

The ELISA was performed on the grains from T_2_ plants to quantify the relative amounts of the avidin protein in each of the six transgenic events (Table 2).

**Table 2:** ELISA readings for the six transgenic events AVD1-AVD6 as well as the blank, negative control (−ve) and positive control (+ve) samples.

Sample	ELISA reading (absorbance)
Blank (Extraction buffer)	0.1
Authentic avidin (+ve)	2.0
Giza 168 (−ve)	0.4
AVD1	1.5
AVD2	0.8
AVD3	0.8
AVD4	1.2
AVD5	1.4
AVD6	1.4

AVD1, AVD5 and AVD6 had the highest absorbance values which refer to the highest avidin protein concentration (1.5 and 1.4), then AVD4 (1.2) and finally AVD2 and AVD3 giving the lowest absorbance indicating low avidin concentration (0.8).

### Insect Toxicity Assay

All insects of the *S. granarius* died after 21 days of feeding on a diet consisting of crushed seeds of transgenic avidin wheat, whereas, none of the tested insects died when they were fed on a diet of ground seeds from non-transgenic wheat (Table 3).

**Table 3:** Means for survival of *Sitophilus granarius* that were fed on the transgenic events and the non-transgenic cultivar crushed seeds in their diets for 21 days.

line	0 day	7 days	14 days	21 days	Mean
AVD1	100a	69d	29 g	0 h	49.5
AVD2	100a	74c	32e	0 h	51.6
AVD3	100a	73c	32e	0 h	51.5
AVD4	100a	70d	30 fg	0 h	50
AVD5	100a	69d	31ef	0 h	50.25
AVD6	100a	70d	31ef	0 h	50.25
G168	100a	100a	98a	91b	97.5
Mean	100	75.3	40.6	13.04	

Means within rows and/ or columns followed by the same letter (s) are not significantly different by Duncan’s new multiple range test (P < 0.05).

## Discussion

Wheat is an important staple crop in many countries. Depending on the climatic and storage conditions, it can become infested by a wide variety of stored-product insect pests [1]. Avidin-containing wheat and its processed products would be resistant to infestations caused by all of the species. Detection and control methods for stored product insects have to be through integrated pest management program (IPM). Major efforts involving sanitation practices, exclusion techniques, habitat modifications, fumigation, and insecticide applications are usually required to prevent damage. The conventional insect control method is mainly dependent on the intensive and extensive use of chemical pesticides, which have drawbacks such as doing harm to the ecological system, producing residual poisons to human beings and animals, and high cost. Moreover, some insects have developed resistance to some of the available insecticides [32,33]. Therefore, it is desirable to develop insect-resistant plants through the introduction of insect-resistant genes (e.g. avidin) through genetic transformation. In present study, synthetic avidin coding DNA was transferred to wheat plants. Avidin accumulation was detected in transgenic plants by ELISA and western dot blot. We found that avidin wheat has excellent resistance to storage insects. Bioassay experiments proved that insect mortality in the first week was about 30 %, in second week it was about 70 % and in the third week the mortality was 100 %. Similar results were obtained with the red flour beetle *Tribolium confusum*, and flat grain beetle *Cryptolestes pusillus* [19]. Avidin transgenic tobacco halted growth and it caused mortality in larvae of two lepidopterans, *Helicoverpa armigera* and *Spodoptera litura*. The insects showed very poor growth over their first 8 days on a diet consisting of the leaves from transgenic plants and significant mortality were reported after 11 or 12 days and all insects were dead after 22 days [34]. In conclusion, the stable avidin transgenic wheat showed high level of resistance to the stored product insect (*Sitophilus granarius*). This study will hopefully decrease the loss of wheat seeds in warehouses significantly. In addition, the avidin-transgenic wheat powder can be used as a bioinsecticide. Avidin may interfere with enzymes that depend on enzyme bound biotin, such as those involved in carboxylation, decarboxylation, and transcarboxylation reactions [35]. Biotin deficiency in the blowfly, *Aldrichina grahami*, caused decreases in several fatty acids [36]. Presumably, a similar biochemical effect led to the stunted growth and mortality of the stored-product insects studied here. The public acceptability of avidin wheat as a food or feed is difficult to predict. Careful examination of its safety, however, is needed before consumption by humans and livestock can be considered. Kramer et al., [19] reported that at least there is no acute toxicity of avidin when feed to mice. Long-term ingestion of high levels of avidin maize may be a problem, because a biotin deficiency can decrease the growth rate of mice and affect reproduction [36,37]. However, avidin is a food protein that is consumed in the form of egg at a concentration of >400 part per million (ppm) by dry weight, which is four times higher than its concentration in most of the wheat used in the present study. Moreover, avidin has an antidote (biotin), which can be used to prevent toxicity or to rescue potential victims from adverse effects. Food and feed uses of avidin wheat might involve processing that includes supplementation with the vitamins. Another method that would help to prevent potential toxicity of the avidin wheat is the heat treatment, which would denature most of the avidin as well as the avidin–biotin complex and release most of the vitamins [38–40]. Currently development of wheat expressing transgenic avidin as a food or feed grain could be considered after thorough risk assessment. In addition to its efficacy against postharvest insect pests, avidin also is effective against preharvest pests such as the beet armyworm, black cutworm, bollworm, and other species for which biotin is an essential growth factor [41,42]. Transferring the avidin gene to other crops will be important in determining its potential usefulness in a variety of other commercial protein production and pest control situations. Risk assessment of these transgenics can be done following the National Biosafety Committee guidelines for the most efficient avidin-transgenic line. In addition, different species of stored cereals insects can be challenged with the avidin transgenic wheat grains and flour.

## Conclusion

A synthetic avidin gene was introduced into spring wheat (*Triticum aestivum* L.) cv. Giza 168 using a biolistic bombardment protocol. Functional integrity of avidin was confirmed by insect bioassay. The results of bioassay using transgenic wheat plants challenged with wheat weevil revealed 100 % mortality of the insects reared on transgenic plants after 21 days. In conclusion, the stable avidin transgenic wheat showed high level of resistance to the stored product insect (*Sitophilus granarius*).

This study will hopefully decrease the loss of wheat seeds in warehouses significantly.

## Abbreviations

ELISA: Enzyme Linked-Immunosorbent Assay
G168: Giza 168
ppm: part per million
SqRT-PCR: Semi-quantitative Reverse transcriptase-polymerase chain reaction
